# Can immunotherapy be useful for a « functional cure » of HIV infection?

**DOI:** 10.1186/1742-4690-8-S2-O33

**Published:** 2011-10-03

**Authors:** Guido Vanham

**Affiliations:** 1 Institute of Tropical Medicine, Antwerp, Belgium

## 

Highly active antiretroviral therapy (HAART) alone cannot eliminate HIV from the body and even in patients with undetectable viral load, full recovery of the immune system is not being obtained, with signs of inappropriate "immune activation" and "immuno-senescence". Therapeutic strategies, complementary to HAART, aim at a "functional cure" i.e. control HIV in such a way that it no longer produces harm to the immune system. Anti-latency drugs, anti-inflammatory agents and/or measures to prevent or eliminate concomitant infections may contribute to a functional cure. Immunotherapy represents another possible strategy, which should reverse deficient T cell generation, aberrant differentiation, exhaustion and ineffective HlV-specific T cell responses.

Four major pathways are being attempted:

1. Administration of so called common γ-chain cytokines, including inter-leukin (IL)-2, IL-7, IL-15 or IL-21, which are crucial in T cell generation, differentiation and function. Only IL-2 has been formally evaluated in a phase 3 trial: although it increased CD4 T counts, it failed to improve clinical outcome.

2. Blockade of negative regulator receptor-ligand interaction, including CTLA-4, PD-1, TIM-3 has been shown to improve HlV-specific T cell function in vitro, but seemingly discrepant results have been obtained in the SIV macaque model. PD-1 blockade lowered viral load and improved survival, whereas CTLA-4 blockade increased viral load, probably because of immune activation.

3. Therapeutic immunization with HIV antigens. Older trials with de-enveloped HIV particles (Remune^®^) failed to improve clinical outcome in HIV-infected subjects. More recent trials, using ALVAC^®^, a canarypox vaccine, containing HIV antigens-encoding genes, mitigated viral load rebound after HAART interruption in several trials, but had no effect or even increased rebound in other trials, the latter result being probably linked to inappropriate immune activation.

4. The most recent tendency is to target dendritic cells with either inactivated autologous virus or virus antigen-encoding RNA. Safety and ímmunogenicity of this strategy has been well established, but clinical results are equivocal as yet. In any case, this approach is amendable to further refinement, with regard to inclusion of RNA encoded patient-adapted viral sequences and co-stimulatory molecules, as well as improved targeting.

In conclusion, several HIV immunotherapy strategies are being attempted, with varying success. The basic challenge remains to find a (combined) strategy, successfully activating effective HlV-specific T cell responses, while reducing inappropriate HIV-promoting immune activation.

